# Resilient Properties of Soil-Rock Mixture Materials: Preliminary Investigation of the Effect of Composition and Structure

**DOI:** 10.3390/ma13071658

**Published:** 2020-04-03

**Authors:** Junfeng Qian, Yongsheng Yao, Jue Li, Hongbin Xiao, Shenping Luo

**Affiliations:** 1 School of Civil Engineering, Central South University of Forestry and Technology, Changsha 410004, China; qianjunfeng@csuft.edu.cn (J.Q.); T20090169@csuft.edu.cn (H.X.); 18679742106@163.com (S.L.); 2 Engineering Research Center of Catastrophic Prophylaxis and Treatment of Road & Traffic Safety of Ministry of Education, Changsha University of Science & Technology, Changsha 410114, China; lijue1207@stu.csust.edu.cn

**Keywords:** subgrade, soil-rock mixture, resilient property, mesostructure, discrete element method, rock content, anisotropy

## Abstract

The physical composition and stress state of soil-rock mixture (SRM) materials have a crucial influence on their mechanical properties, and play a vital role in improving the performance of subgrade. To reveal the resilient behavior and mesostructure evolution of SRM materials, triaxial tests and discrete element method (DEM) numerical analysis have been carried out. In the triaxial test section, the mechanical response of SRM materials was investigated by preparing samples under different stress states and physical states and conducting triaxial tests on samples. Simultaneously, a new irregular particle modeling method was developed and applied to the discrete element modeling process to analyze the mesostructure evolution of SRM materials under cycling loading. First, a cyclic triaxial test of SRM material is performed on the SRM material, and the effects of bulk stress, octahedral shear stress and rock content on the resilient modulus of the SRM material are analyzed. It is revealed that the resilient modulus increases with increasing bulk stress and rock content, and decreases with increasing octahedral shear stress. Based on a new resilient modulus prediction model, the relationships among the rock content, stress state and resilient modulus are established. Then, based on an improved DEM modeling method, a discrete element model of the SRM is established, and the influence of rock content on coordination number and mesostructure evolution of the SRM is analyzed. The results show that in SRM materials, the increase of crushed rock changes the mesostructure of the SRM material. With the increase of rock content, the internal contact force changes from “between soil and rock” to “between rocks”, and the skeleton formed in the rocks gradually develops overall stiffness. Under the condition of low stress, the anisotropy of the SRM material is mainly caused by the shape and grade distribution of crushed rock. The induced anisotropy caused by the change of stress state has little effect on its mechanical behavior, which may lead to the greater dispersion of multiple SRM test results.

## 1. Introduction

With the development of China’s infrastructure construction, it is inevitable that more and more hydraulic, traffic and tunneling structures are built on or in the engineering geological body of the soil-rock mixture (SRM) [1]. SRM is a general term for a range of mixed geomaterials, which contains many coarse-grained rocks embedded within fine-grained soils, and is widely distributed in nature [2]. However, there is a clear gap between the rock blocks and soil matrix in particle size and material composition, so it shows two extreme characteristics in the mechanical properties, that is, the rock mass is highly rigid, while the soil particles are soft and hard. Due to the heterogeneous structure of SRM, its mechanical properties depend on these structural characteristics (compositions, density, strength, etc.) and degree of bonding in the matrix, resulting in a significant difference from natural rocks or soils. Furthermore, other rock characteristics, such as rock content (*R_c_*), shape, porosity, gradation and distribution, also contribute significantly to the deformation mechanism and anisotropic behavior of granular materials [3,4].

Many early experimental investigations were conducted to focus on shear behaviors and its influencing factors of SRM, which can be categorized in two aspects: composition and structure. On the one hand, rock content is an important index in evaluating material composition, which has received extensive attention and research [5]. After synthesizing intensity characteristics of SRM in multiple regions, researchers have pointed out that when the *R_c_* exceeds a certain threshold, the macro strength of SRM increases with the increase of *R_c_* [6,7]. Miller et al. conducted a triaxial compression test using a mixture of clay and coarse sand, and found that when the *R_c_* increased to 50% to 70%, the internal friction angle would increase sharply and the cohesive force would decrease [8]. Similarly, Xu et al. considered that the *R_c_* of 30% and 70% are two characteristic points that affect the macroscopic performance of SRM, and the results from shear tests showed that it is an obvious increasing trend for the internal friction angle with the increase of the *R_c_* [9]. On the other hand, some studies have attempted to analyze the particle composition and distribution of SRM using digital image technology (DIT) and found that the non-uniform and discontinuous characteristics of its mesostructure have a significant impact on its mechanical response to mechanical properties through mechanical tests [10,11]. Wang et al., combined with X-ray CT scanning technology, found that the relative position of rock blocks has a great influence on the stress distribution of SRM, especially at the interface of rock and soil [12]. Dondi [13] and Cavarretta et al. [14] analyzed the effects of aggregate shape, angularity and size distribution on the characteristics of the filler in a triaxial test. They held that the increased angularity of the particles helps the interlocking effect better.

In flexible pavements, the resilient behavior of SRM plays a key role on the performance of pavement structure. SRM is used extensively as a cheap and abundant roadbed filler, which provides sufficient stiffness, strength and stability for high-quality pavement structure carrying their own weight and traffic loads [15]. The resilient modulus (*M_R_*) of subgrade materials has been employed by the current mechanistic-empirical pavement design guide (MEPDG) [16] as the primary mechanical parameter for calculating the dynamic deflection of its top surface; the deflection is widely considered as the main factor to cause early pavement damage, such as rutting and cracking [17]. The resilient modulus is defined as the ratio of cyclic deviatoric stress to relative elastic strain in the Repeated Load Triaxial (RLT) test [18]. The recent researches on resilient behavior are always dependent on the approach of phenomenology, which requires a large number of physical model tests and statistical methods to reveal the changes in its mechanical property [19]. In fact, with limited test conditions, the results obtained in the laboratory test may differ greatly from the actual engineering [20]. Additionally, the complexity and randomness in the mesostructure of SRM are difficult to be accurately described by the continuum mechanical model [21]. Therefore, in recent years, some general mesostructural approaches and prediction models have been proposed to describe the resilient behavior of unbound granular materials [22,23,24]. These models establish the relationship between the evolution of meso parameters and actual engineering characteristics. However, few attempts to study SRM have been proposed.

To accurately describe the resilient characteristics of the soil-rock mixture, the purpose of this study is to carry out laboratory tests to evaluate the resilient characteristics of SRM considering the effect of rock content, and to establish a numerical model to analyze the meso-mechanism of the crushed rocks related to the resilient behavior and anisotropy. The structure of this paper is as follows. In Section 2, it introduces material properties and test methods used to determine the resilient the resilient modulus. Then, experiment results are presented in Section 3, and a new prediction model for calculating the SRM resilient modulus considering different rock contents is proposed through a statistical method. After that, the improved discrete element method (DEM) model in Section 4 is used to model and carry out a series of numerical tests, in order to clarify the influence of material mesostructure on the resilient behavior. The numerical test results are used to calculate the stress-strain response, interlock characteristics (coordination number and C value) and to discuss the induced anisotropy evolution under different loading stages and rock content in Section 5. Finally, some findings are summarized in the last section.

## 2. Sample Preparation and Testing Method

### 2.1. Materials and Sample Properties

The soil used in this study is a typical granite residual soil taken from K117 + 200 of the Guangzhou-Foshan Expressway in Guangdong Province, China. It was identified as the low liquid limit clay, and its physical properties are shown in Table 1. Through compaction tests in the laboratory, the optimum water content and maximum dry density were determined to be 10.5% and 1890 kg/m^3^, respectively. The tested rock block is limestone crushed rock which comes from a crushed rock factory near the construction field of the Guangzhou-Foshan expressway. Its bulk density is 2750 kg/m^3^.

For the physical properties of SRM, the rock content (*R_c_*) has been a commonly measured structure index, which is important in determining the mechanical response [25]. In addition, some limits of the relative concentrations of rock and soil in strength control of SRM exist, as introduced by Vallejo et al. [7]. Through laboratory tests, they found that when the concentration by mass of rock blocks is >75%, the mechanical performance of SRM has approached that of the pure rock mixture. If the *R_c_* is <30% by mass, the strength of SRM is mainly dominated by the soil and has little effect on the increase of rock concentration [26]. Therefore, the research object in this study is the mixture with the *R_c_* between 30% and 75%. Five mass fractions of crushed rock, 30%, 40%, 50%, 60% and 70% were selected for different rock content experiments.

The rock is mixed into the soil sample, and the compaction test is carried out to obtain the change law of the optimal moisture content and the maximum dry density, as displayed in Table 2. According to the recommendations of NCHRP 1-28A [27], when the maximum particle size of the sample does not exceed 19 mm, a test piece with a diameter of 100 mm should be selected. Combined with the test equipment of the National Key Engineering Laboratory of Changsha University of Science and Technology, this study chooses 9.5–19 mm SRM test piece made from crushed rock and soil less than 2 mm. The size of the testing specimen is 100 mm × 200 mm.

The moisture content of subgrade will be controlled as optimal moisture content during road construction. Since this article simplifies the effect of moisture content on the test piece, it only considers the resilient modulus of SRM under the optimal moisture content state. According to the requirements of China Highway Subgrade Design Code, field control standards generally have 90%, 93% and 96% compactness. The resilient properties of soil-rock mixtures with different compactness basically correspond to that of rock properties. To facilitate analysis and discussion, a 96% compacted specimen is selected for RLT test. Therefore, to ensure the consistency of the samples, the standard routine preparation procedures were used to prepare SRM with different rock contents (30%, 40%, 50%, 60%, 70%), and soil samples with 0% rock contents were used for comparison, and the SRM was prepared in advance according to the calculated moisture content. Vibration forming method is selected for the forming of samples to ensure that each sample has the same degree of compaction. During the forming process, the rock content of each layer should be the same and compacted. After reaching the relative density, add the next layer of material until it is finally complete.

### 2.2. Repeated Load Triaxial (RLT) Testing Program

A test loading sequence must be identified to investigate the SRM resilience under different stress conditions. Many previous studies have proposed different sequences, such as AASHTO T307-99 [28] and NCHRP 1-28A [27]. Based on the working conditions of the subgrade in the southern region of China, the loading sequence of the RLT test in this study was selected as shown in Table 3, as proposed by Zhou [29]. This sequence is obtained by collecting and calculating the stress states of 10 typical highway subgrades in South China. It is found that the stress range of the subgrade is 30~50 kPa and the deviator stress of the top of the subgrade is less than 68 kPa under 50% overload. At the same time, the failure of unbonded material is greatly affected by deviatoric stress. Therefore, in combination with the above analysis, the loading sequence in Table 3 is appropriate, which covers the general stress states of the subgrade and ensures that the test specimens are not easily damaged.

Half-sinusoidal loading pulse cyclic loading for 0.2 s duration and 0.8 s rest time was used in the test. Then, 40 kPa cell pressure and 30 kPa deviator stress were selected to load 2000 cycles to eliminate plastic deformation before formal loading. Each sequence is loaded for 100 cycles during formal loading. The resilient modulus is calculated by selecting the last 5 cycles of 100 loading cycles in each stress state, according to Equation (1).

(1) $$M_{R}=\frac{\sigma_{d}}{\varepsilon_{r}}=\frac{\sigma_{1}-\sigma_{3}}{\varepsilon_{r}}$$

where *M_R_* is the resilient modulus; *σ_d_* is the deviator stress; *ε_r_* is the resilient strain, *σ*_1_ is the axial cyclic stress; and *σ*_3_ is the cell pressure.

## 3. Effect of Stress Condition and Rock Content

### 3.1. Analysis of Experiment Results

Similar to the analysis method of subgrade soil, stress state and material composition have an essential influence on the resilient modulus of SRM materials. To predict the resilient modulus, many models have been proposed, which are based on the stress state. The most classic model is NCHRP1-28A, which is a three-parameter model. The model can predict the resilient modulus of materials based on the stress state, according to Equation (2).

(2) $$M_{R}=k_{1}P_{a}{\left(\frac{\theta }{P_{a}}\right)}^{k_{2}}{\left(\frac{\tau_{\mathrm{oct}}}{P_{a}}+1\right)}^{k_{3}}$$

where *M_r_* = resilient modulus; *θ* is the bulk stress, *θ* = *σ*_1_ + *σ*_2_ + *σ*_3_, *σ*_1_ is the major principal stress, *σ*_2_ is the intermediate principal stress, *σ*_3_ is the cell pressure; *τ_oct_* is the octahedral shear stress. The transformation from octahedral shear stress to deviator stress is $\tau_{oct}=\frac{\sqrt{2}}{3}q_{cyc}$; *P_a_* is standard atmosphere pressure, and it is 101.325 kPa by default; *k*_1_, *k*_2_*, k*_3_ are the regression coefficients.

**Figure 1:**
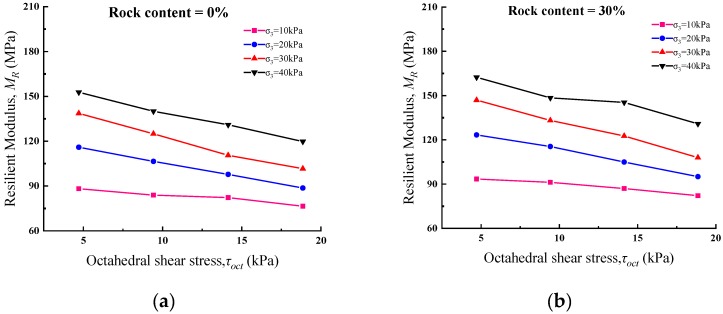

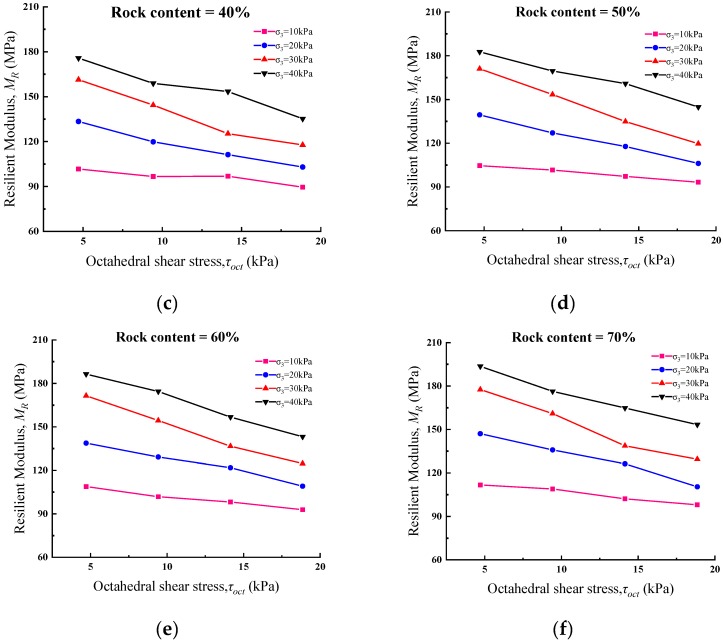
Relationship between SRM resilient modulus and octahedral shear stress (Compaction= 96%) with different *R_c_*: (**a**) 0%; (**b**) 30%; (**c**) 40%; (**d**) 50%; (**e**) 60%; (**f**) 70%.

Octahedral shear stress and bulk stress in NCHRP1-28A model are considered as indexes that can reasonably reflect the stress state of the specimen. Therefore, these two stress indexes are also used in this study to discuss the relationship between the resilient modulus and the stress state of the specimen. Figure 1 shows the relationship between octahedral shear stress and resilient modulus at different rock contents under 96% degree of compaction. As shown in Figure 1, the resilient modulus decreases rapidly with increasing octahedral shear stress at 0% to 70% rock contents. For example, under 40 kPa cell pressure, when the octahedral shear stress increases from 4.71 kPa to 9.43 kPa, 14.14 kPa and 18.85 kPa, the resilient modulus of the soil specimen decreases by 8.3%, 14.2% and 21.6%, respectively. On the other hand, for the sample with 60% rock content, when the resilient modulus value increases from 4.71 kPa to 9.43 kPa, 14.14 kPa and 18.85 kPa under 40 kPa cell pressure, the resilient modulus value of the soil rock mixture decreases by 6.4%, 15.9% and 23.2%, respectively. This regulation shows that the influence of octahedral shear stress on soil specimen and SRM specimen is similar, and the soil rock mixture has significant stress dependence under cyclic load.

**Figure 2:**
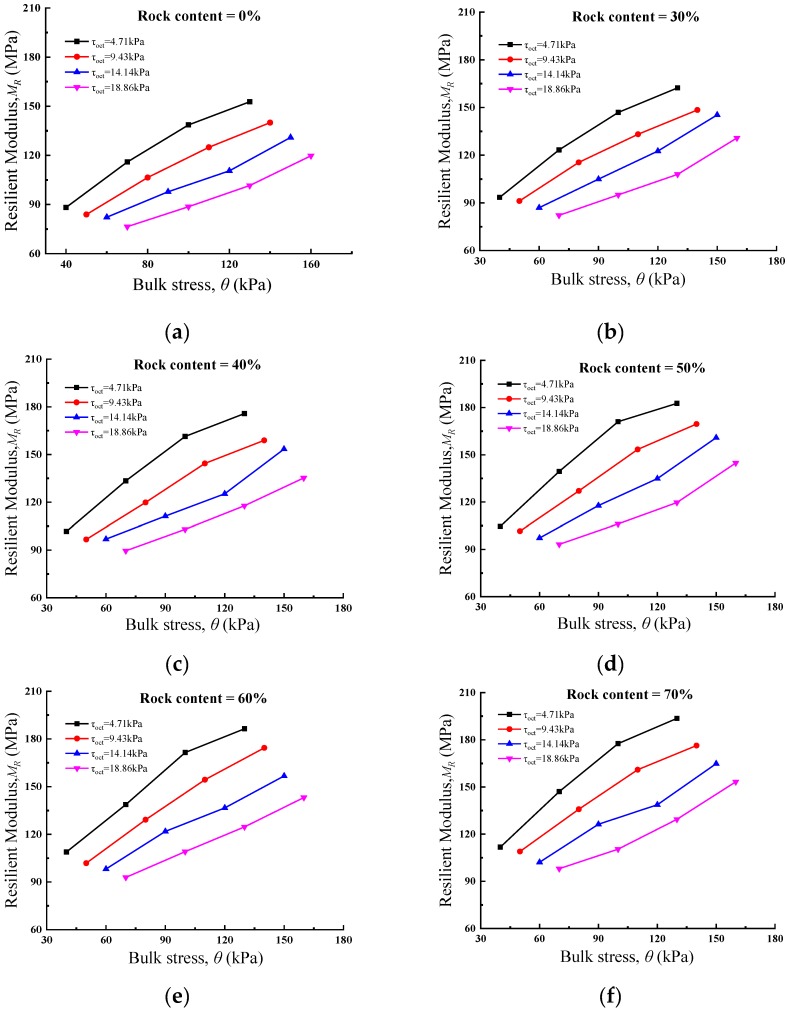
Relationship between bulk stress and resilient modulus with different *R_c_*: (**a**) 0%; (**b**) 30%; (**c**) 40%; (**d**) 50%; (**e**) 60%; (**f**) 70%.

Bulk stress describes the stress of an object in space along the three directions X, Y and Z, which can characterize the stress state of material in space effectively. Figure 2 demonstrates the relationship between bulk stress and resilient modulus for different rock contents. From the test results, it can be seen that under the same octahedral shear stress, the resilient modulus of soil rock mixture specimens increases with the increase of bulk stress, which is also reflected on the soil specimens. For instance, when the rock content of the specimen is 70%, and the octahedral shear stress is 4.71 kPa, the corresponding resilient modulus values are 111.75 MPa, 147.05 MPa, 177.55 MPa and 193.61 MPa when the bulk stress is 40 kPa, 70 kPa, 100 kPa and 130 kPa, respectively. This result reflects that the bulk stress acts as a restraint on the specimens, which on the specimens, has a curing effect and thus increases the overall stiffness of the material.

### 3.2. Evaluation for the Effect of Rock Content on Resilient Properties

**Figure 3:**
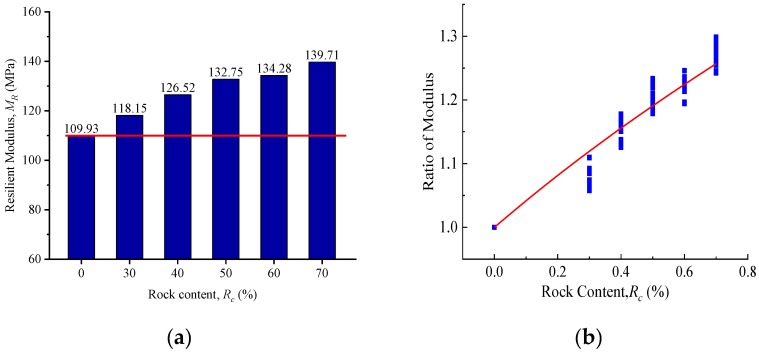
Parametric variation with rock content: (**a**) resilient modulus; (**b**) ratio of modulus.

Using the average resilient modulus to evaluate the influence of different rock content on SRM materials can reflect the relationship between rock content and average resilient modulus. Figure 3a shows the relationship between average resilient modulus and rock content. *R_c_* is used to represent the mass of rock in the specimen divided by the total mass of the specimen. It can be seen from the figure that, from 30% to 70% rock content, comparing the soil sample (0% rock content), the resilient modulus of SRM increased 7%, 15%, 20%, 22% and 27%, respectively. Meanwhile, to eliminate the influence of stress state on the results, the ratio of SRM’s modulus to soil’s modulus under different rock content is used to characterize the influence of rock content on SRM’s material resilience performance, and the normalization treatment is carried out. It is found that with the increase of rock content, the increasing trend of resilient modulus is approximately linear, as shown in Figure 3b.

According to the above analysis, the material properties of SRM are mainly affected by the rock content. Therefore, to quantitatively analyze the influence of rock content on the modulus value, adding *R_c_* to the NCHRP 1-28A model to modify the predicted model can help to present the resilient modulus of SRM material. Considering the influence of rock content on SRM material, in the new prediction model, the physical parameter rock content is put in the first item to characterize the effect of physical state in SRM material on the result of resilient modulus.

**Figure 4:**
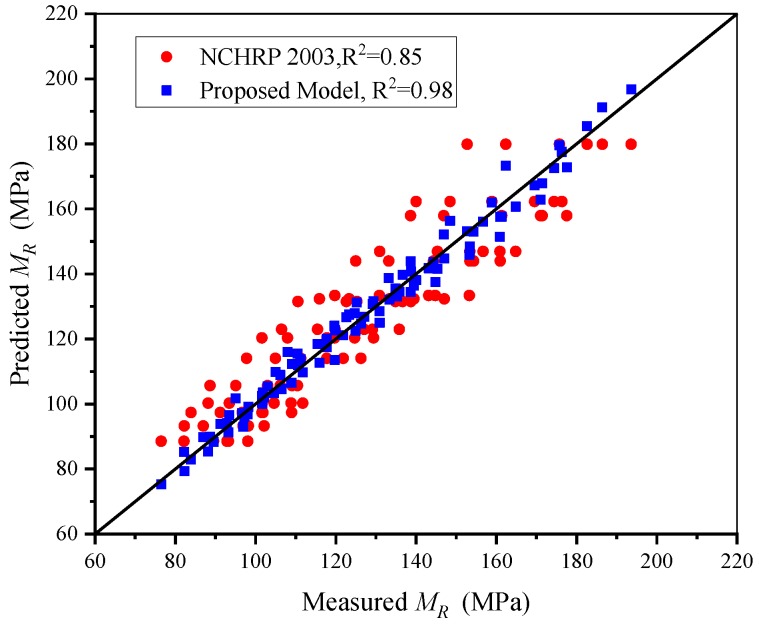
Comparison of the proposed model and the NCHRP 1-28A model.

The results in Figure 3b show that there is a critical point in the process of increasing the rock content, which makes the increase of modulus change from rapid increase to moderate increase. Therefore, the rock content added in the model cannot be characterized by linearity, and the changing trend of average modulus also reflects this phenomenon. The first item is expressed in the form of (*R_c_* + 1), which can characterize its relationship with *M_R_*. Referring to the establishment of the NCHRP 1-28A model and the above experimental results, the second and third items are respectively constructed by volume stress and octahedral shear stress, and their forms are exponential. The regression coefficients *k*_1_, *k*_2_, *k*_3_ and *k*_4_ are used to reflect the relationship between each term in the model and the resilient modulus. Finally, the new model is established as Equation (3). The newly proposed model comprehensively reflects the stress state, shear effect and physical state, so it can reasonably estimate the resilient modulus value of SRM materials. The parameters and accuracy of the model are shown in Table 4. Comparing the measured and estimated resilient modulus, and the new model has been compared to the NCHRP 1-28A model, as shown in Figure 4. From the comparison results, the new model has higher prediction accuracy, which indicates that the newly proposed model can provide some guidance for the research of subgrade mixed materials.

(3) $$M_{R}=k_{1}P_{a}{\left(R_{c}+1\right)}^{k_{2}}{\left(\frac{\theta }{P_{a}}\right)}^{k_{3}}{\left(\frac{\tau_{\mathrm{oct}}}{P_{a}}+1\right)}^{k_{4}}$$

From the above test results, it can be concluded that *R_c_* has a significant impact on the physical properties of SRM materials, which will cause the material stiffness to change with the change of *R_c_*. In the next part, the evolution of mesoscopic behavior in SRM is analyzed by numerical simulation of the DEM model.

## 4. Numerical Models Based on Rock Morphology

The macro-mechanical response of geotechnical materials is a comprehensive manifestation of the interaction (friction, deformation, motion, etc.) between particles under the action of external forces, where the shape of the particles plays an important role [30]. Previous studies have shown that as the particle irregularity increases, the porosity of the test specimen decreases, the sensitivity to stress increases, and the stiffness of the test specimen increases through interlocking [31]. Therefore, it is essential to simulate the resilient behavior and anisotropic evolution of SRM by constructing irregular aggregates.

### 4.1. Methodology on Morphology and Meso-Structure

**Figure 5:**
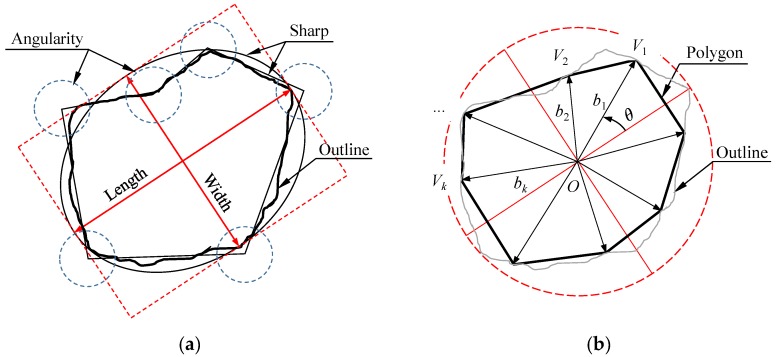
Description of particle morphology: (**a**) rock outline; (**b**) irregular polygon.

In the morphological analysis of particles, the aspect ratio (*A_r_*) and Roundness (*R*) are two widely used quantitative parameters to describe the particle profile, as shown in Figure 5a. The axial ratio is defined as the ratio of the major and minor axis lengths of the particle equivalent ellipse, as shown in Equation (4), which is a crucial parameter reflecting the particle size distribution; roundness is an important parameter that indirectly characterizes the shape and angularity of the particle, calculated by Equation (5). When the roundness is 1.0, the particles are perfectly round.

(4) $$A_{r}=\frac{d_{L}}{d_{W}}$$

(5) $$R=\frac{P^{2}}{4\pi A}$$

where *d_L_* and *d_W_* stand for the length and width of the equivalent ellipse in the 2D plane; *P* and *A* stand for the perimeter and area of the particle outline. 

In theoretical research, researchers generally think that it is reasonable and effective to simulate the shape of particles with an approximate ellipse or polygon, and have obtained some meaningful conclusions and methods [32]. However, the particles in these methods are randomly generated by the computer and do not depend on the characteristics of the real aggregate, which leads to some inevitable defects and difficulties in numerical modeling and parameter determination [33]. For example, in Figure 5b, the contours of real particles have some concave defects instead of completely convex polygons, which is more conducive to the use of the intergranular interaction between particles. In addition, it is not described that as the number of vertices that generate a polygon is larger, the more accurate the particle shape. In previous investigations and studies, we found that the number of approximate polygon vertices of real particles has a certain regularity, obeys the normal function distribution and is mainly concentrated between 4 and 8 [34]. Therefore, it is necessary to develop an irregular shape particle simulation method based on the morphological characteristics of real rocks.

For the mesostructure of the mixture, its evolution mechanism cannot be separated from a basic unit, the contact point, which provides a way of stress transfer between the particle skeleton. According to the research of Shi et al. [10], coordination number (*n_c_*) and *C* value are used to evaluate the occlusion characteristics of coarse particles in SRM. The calculation formulas are Equations (6) and (7). At the same time, under the action of external force, the distribution and stress state of particles change, which is directional in probability density and is called induced anisotropy. In this study, the induced anisotropy of SRM during resilient deformation is also analyzed.

(6) $$n_{c}=\frac{N_{c}}{N_{p}}$$

(7) $$C\;\mathrm{value}=1-\frac{A_{c}}{A_{r}}$$

where *N_c_* and *N_p_* stand for the total number of contacts and the particle number; *A_r_* and *A_c_* stand for the total area of rock blocks in SRM and the area of rock blocks which are in contact with other rocks. 

### 4.2. Irregularly Shaped Clumps and Contact Models

For the generation and assembly of irregular particles, Zhang et al. [35] proposed a geometric anisotropy modeling method, which can perform irregular shape clump filling under the premise of ensuring the uniformity of the initial test. However, the generated blocks are all convex polygons, while most of the natural rock blocks are convex and concave polygons. For this reason, based on Zhang’s research, this study improves the method for determining the coordinates of the vertices of the rock, see Equation (8). At the same time, a roughness coefficient (*C_r_*) is introduced to control the degree of unevenness, which is defined as the ratio of the minimum value *b*_min_ of the vertex ray length to the average value *b*_A_. It can be known from geometric theory that when *C_r_* is close to cos (2π/*n*_v_), the generated particles are more likely to be concave polygons. The statistical simulation results show that the average particle size of the newly generated particles is about 0.77 times the initial particle size, so the original particles need to be enlarged by a certain proportion to keep the original particle gradation consistent.

(8) $$\{\begin{array}{l} x_{k}=x_{0}+b_{i}\cos\theta_{k}\\ y_{k}=y_{0}+b_{i}\sin\theta_{k} \end{array}$$

where *x*_0_ and *y*_0_ stand for the position of the center point (*O*) in Figure 5b; *x_k_* and *y_k_* stand for the vertex position; *k* stands the ID of the vertex in the range from 1 to *n_v_*; *b_i_* is the length of the reference ray, which follows the uniform distribution from 0.6 to 1.0 of the radius of the equivalent circle.

In the discrete element model, the mesoscopic parameters of the particles are reflected by the contact model. Before choosing a contact model, researchers need to distinguish three types of contact interfaces in SRM: contact between rocks and rocks, contact between rocks and soil and contact inside soil particles. Linear model (LM) provides a linear friction component, which can be used to simulate the occlusal contact force between rocks; since the Linear contact bond model (LCBM) can withstand tensile strength, it is used in the mechanical performance characterization of mixtures involving soil particles. The contact of the soil-rock interface adopts LCBM and attribute inheritance, and its mesoscopic parameters depend on the stiffness of the rock and soil. Based on the laboratory test results and related literature, we have listed the mesoscopic parameters corresponding to these interfaces and contact models, as shown in Table 5.

## 5. Meso-Structural Evolution on Resilient Behavior

### 5.1. Contribution of Rock Content to Contact Characteristics

**Figure 6:**
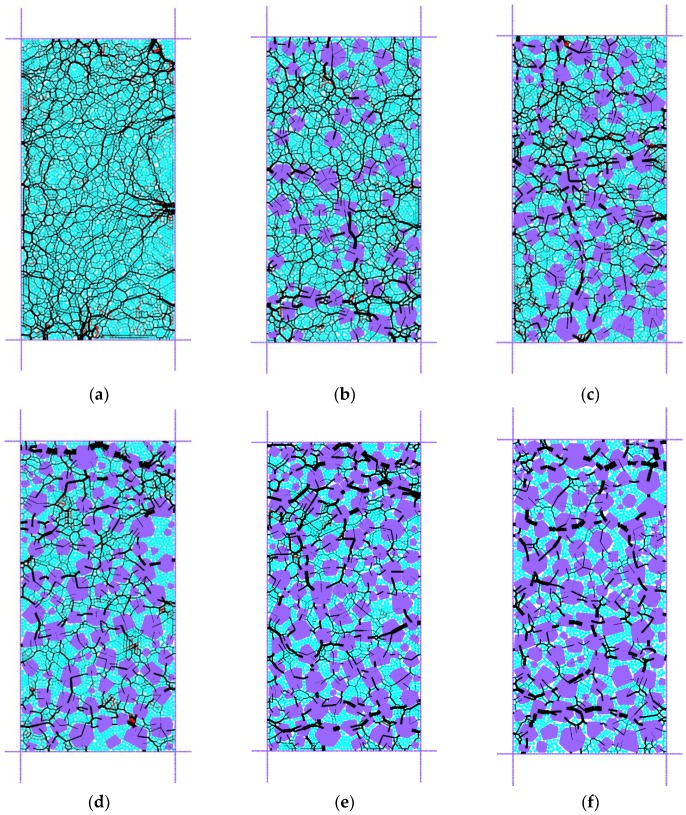
Contact-force distribution of DEM models at the 40kPa cell pressure with different *R_c_*: (**a**) 0%; (**b**) 30%; (**c**) 40%; (**d**) 50%; (**e**) 60%; (**f**) 70%.

To intuitively understand the effect of rock content on the mechanical behavior of SRM, Figure 6 shows the contact force distribution for different rock content numerical specimens, in which the specimens are in equilibrium under a cell pressure of 40 kPa. Two-color lines are used to indicate the two types of stress. The pressure is black, and the tension is red. The thickness and orientation of the line through the contact point are used to indicate the relative magnitude and normal direction of the contact force. From Figure 6, it can be seen that the mesostructure of the SRM found significant changes. When the *R_c_* is 0%, the contact force distribution inside the soil sample is uniform, and some major contact forces are concentrated on the contact surface with the wall. As more rocks are added, their contact force tends to be distributed between the rocks and the surrounding soil. When the *R_c_* is 30 %, the load on the specimen is shared by the soil and the gravel, and the interlocking effect of the gravel is not obvious; when the *R_c_* is 40%, there is a clear path between the gravels from below. The external force is transmitted upward to the inside of the test piece; when the *R_C_* reaches 50%, this is obvious. The test piece resists external stress through the contact between the gravel bodies, and the occlusion skeleton between the gravels is initially formed. When the *R_c_* is 60–70%, the crushed rock skeleton plays a leading role in the mechanical response of the specimen, because the contact force between the soil particles is much smaller than the contact force between the crushed rocks. Additionally, the tensile force mainly occurs in the soil medium. With the increase of *R_c_*, the inside of the specimen mainly shows compression characteristics.

**Figure 7:**
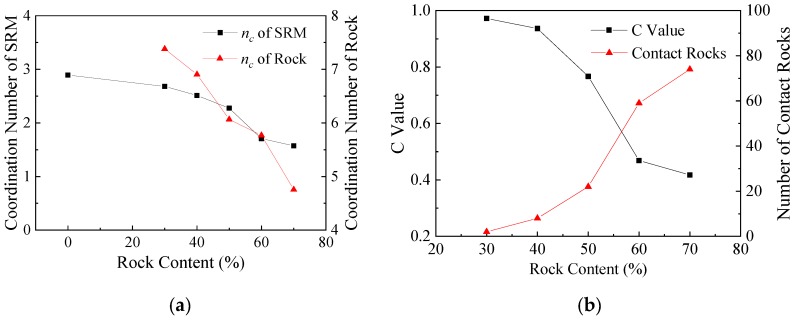
Contact characteristics of numerical specimens at 40 kPa cell pressure: (**a**) coordination number (*n_c_*); (**b**) C value.

Coordination number (*n_c_*) and *C* value were used to further quantitatively analyze the influence of rock content on contact characteristics. It can be seen in Figure 7a that the decrease of the coordination number in the test piece is faster with the addition of crushed rocks. This shows that with the increase of the rock content, the contact state of the soil particles in the specimen changes significantly. In addition, the formation of the crushed rock skeleton leads to an increase of the suspended particles in the soil. At the same time, the coordination number of gravel changes also reflects this problem, with the increase of the rock content, the soil particles surrounding the gravel decrease. The *C* value is used to reflect the effect of the crushed rock skeleton. The smaller the *C* value, the closer the interlock between the crushed rock skeletons. It can be seen from Figure 7b that with the increase of the rock content, the number of crushed rock particles increased, and the *C* value gradually decreased from 97.2% to 41.7%. In other words, when the particle content reached 70%, more than half of the particles in the gravel framework contributed to the contact force distribution, which is consistent with the conclusion from Figure 6.

### 5.2. Gradual Development of Coordination Number and Anisotropy

**Figure 8:**
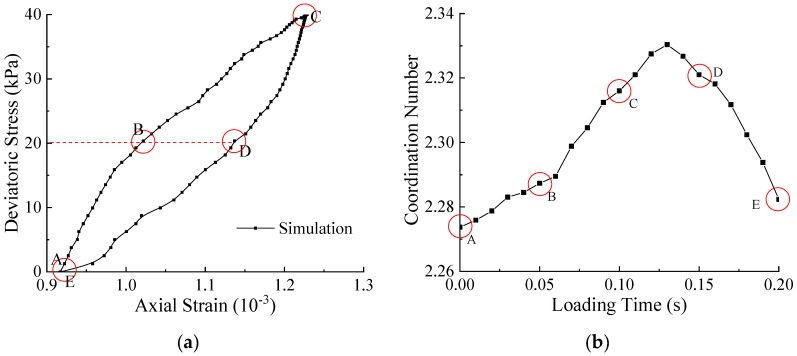
Gradual development with different loading state: (**a**) stress-strain relationship; (**b**) coordination number.

For the same specimen, the change of coordination number indirectly reflects the compaction of the specimen. In this study, an SRM specimen with a rock content of 50% was taken as an example to analyze the mesostructure evolution process under 40 kPa cell pressure and 40 kPa maximum deviator stress. The stress-strain relationship and coordination number evolution during loading are shown in Figure 8. In the loading curve, five characteristic points are selected for research according to deviator stress states, which are: initial state A, intermediate process B, peak state C, resilient process D and final state E, as shown in Figure 8a. The loading time corresponding to these characteristic points is 0.0 s, 0.05 s, 0.1 s, 0.15 s and 0.2 s, respectively, during a half-sinusoidal loading pulse cyclic loading for 0.2 s duration. It can be seen from the evolution of the coordination number of the particles that the compaction degree of the test piece changes dynamically during the loading process. The coordination number increases first and then decreases, and the most compact state of the specimen is not at the peak state C. This shows that there is a certain hysteresis in the bulk strain of the test specimen along with the change of the bias stress, which is consistent with the actual test results and the phenomenon of hysteresis loops.

**Figure 9:**
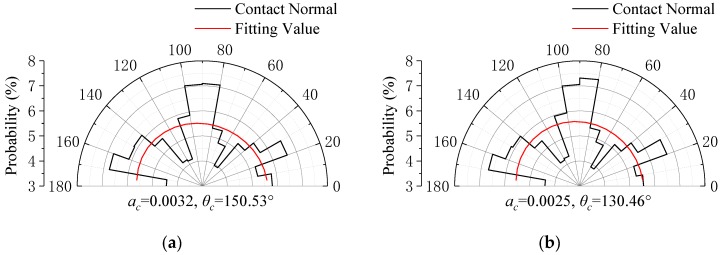

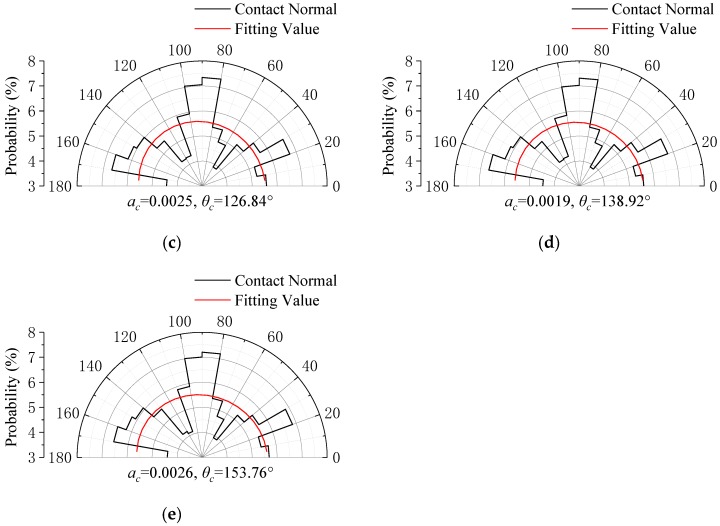
Gradual development of anisotropy: (**a**) initial state A; (**b**) intermediate state B; (**c**) peaking state C; (**d**) resilient process D; and (**e**) final state E.

In the mesostructure of granular materials, its anisotropy is also a fundamental property and has a certain degree of stress correlation. To this end, this study describes the anisotropy of the specimen by measuring the distribution characteristics of the normal direction of contact between particles. It is generally considered that the contact normal distribution *E*(*θ*) satisfies the form of the second-order Fourier function, see Equation (9). Figure 9 shows the anisotropy change during loading. As the bias stress increases, the principal direction angle of the contact normal changes significantly and approaches the principal stress direction of the specimen. From state A to state C, *θ_c_* decreases monotonically; from state C to state E, θc increases monotonically. It can also be seen from the figure that with the increase of deviator stress, the anisotropy coefficient of the test piece has been at a low value and the change is relatively small. At the same time, combined with the anisotropic distribution image, the results show that the anisotropy inside the specimen under low stress depends mainly on the anisotropy of the crushed rock, which is mainly related to the shape and distribution of the particles [36]. However, in triaxial tests, the effect of stress-induced anisotropy is small. Therefore, in the resilient behavior of SRM, it is crucial to consider the structural characteristics of the crushed rock. Further research should consider the influence of shape variability on the performance of the test piece.

(9) $$E(\theta )=\frac{1}{2\pi }\left[1+a_{c}\cos\left(2\left(\theta -\theta_{c}\right)\right)\right]$$

where *a_c_* stands for the anisotropy coefficient of contact normal; *θ_c_* stands for the principal direction angle of contact normal.

## 6. Conclusions

In this paper, the RLT test was performed on SRM materials. The purpose is to reveal the relationship between the rock content and the mechanical properties of the SRM under cyclic loading, and to characterize the rebound performance by resilient modulus, thereby evaluating the mechanical strength of the SRM. Based on the DEM models of the SRM with different rock contents, the mechanical behavior of SRM with different rock contents was studied. The main conclusions are as follows:

1. The specimens under different working conditions were fabricated and suffered cyclic loaded to investigate the effects of stress state and physical state on the resilient modulus. The shear effect of SRM materials is manifested as a stress softening effect. With the increase of deviatoric stress, the resilient modulus of SRM gradually decreases. For SRM materials with different rock contents, under the same compaction condition, the resilience modulus increases with increasing rock content, and decreases with decreasing rock content. Additionally, a prediction model was proposed to describe the influence of rock content and other factors on the resilient modulus of SRM quickly and quantitatively.
2. The addition of crushed rock changes the mesostructure of the SRM material. When the rock content is low (0–30%), there is no rock particle skeleton inside the specimen. At this time, the contact force in the specimen is mainly between the crushed rocks, and between crushed rocks and soil particles, so the external load is shared by the soil particles and crushed rocks. With the further increase of the rock content, the force chains formed between the crushed rocks tend to be perfect, which are mainly used to resist external loads.
3. Through the DEM model, it is found that the results of multiple SRM tests have some discreteness. This is because under low stress condition, the anisotropy of the material is mainly caused by the shape and grade distribution of the crushed rock. This anisotropic behavior leads to a change in coordination number in the material, which affects its contact force. However, the induced anisotropy caused by changes in stress state has little effect on its mechanical behavior.

## Figures and Tables

**Table 1:** Physical properties of granite residual soil in Guangdong.

Water Content (%)	Liquid Limit (%)	Plastic Index (%)	Less than 0.075 mm (%)
22.0	44.0	20.3	51.2

**Table 2:** Results of soil-rock mixture (SRM) compaction tests at different rock contents.

Rock Content (%)	Maximum Dry Density (kg/m^3^)	Optimal Moisture Content (%)
0	1890	10.5
30	1990	7.72
40	2070	6.95
50	2110	6.33
60	2240	5.97
70	2380	4.88

**Table 3:** Triaxial testing sequence for the soil-rock mixture.

Order	Cell Pressure (kPa)	Deviator Stress (kPa)	Major Principal Stress (kPa)	Cycle-Index
0(pre)	40	30	70	2000
1	40	10	50	100
2	40	20	60	100
3	40	30	70	100
4	40	40	80	100
5	30	10	40	100
6	30	20	50	100
7	30	30	60	100
8	30	40	70	100
9	20	10	30	100
10	20	20	40	100
11	20	30	50	100
12	20	40	60	100
13	10	10	20	100
14	10	20	30	100
15	10	30	40	100
16	10	40	50	100

**Table 4:** Regression parameters of the new model.

*k*_1_	*k*_2_	*k*_3_	*k*_4_	*R* ^2^	Correlation
1.5558	0.4731	0.4960	−3.1759	0.98	Excellent

**Table 5:** Mesoscopic parameters and contact models.

Item	Rocks	Soils
Contact Model	Linear Model	Linear Contact Bond Model
Density (kg/m^3^)	2700	2000
Normal Stiffness (GPa)	5	1
Shear Stiffness (GPa)	2.4	0.2
Friction Coefficient	0.9	0.6
Critical Damping Ratio	0.8	0.8
Tensile strength (kPa)	—	4.0
Shear strength (kPa)	—	4.0
